# Characterization of 4-HNE Modified L-FABP Reveals Alterations in Structural and Functional Dynamics

**DOI:** 10.1371/journal.pone.0038459

**Published:** 2012-06-06

**Authors:** Rebecca L. Smathers, Kristofer S. Fritz, James J. Galligan, Colin T. Shearn, Philip Reigan, Michael J. Marks, Dennis R. Petersen

**Affiliations:** 1 Department of Pharmaceutical Sciences, Skaggs School of Pharmacy and Pharmaceutical Sciences, University of Colorado Anschutz Medical Campus, Aurora, Colorado, United States of America; 2 Department of Pharmacology, School of Medicine, University of Colorado Anschutz Medical Campus, Aurora, Colorado, United States of America; 3 Institute for Behavioral Genetics, University of Colorado, Boulder, Colorado, United States of America; University of Cincinnati, United States of America

## Abstract

4-Hydroxynonenal (4-HNE) is a reactive α,β-unsaturated aldehyde produced during oxidative stress and subsequent lipid peroxidation of polyunsaturated fatty acids. The reactivity of 4-HNE towards DNA and nucleophilic amino acids has been well established. In this report, using proteomic approaches, liver fatty acid-binding protein (L-FABP) is identified as a target for modification by 4-HNE. This lipid binding protein mediates the uptake and trafficking of hydrophobic ligands throughout cellular compartments. Ethanol caused a significant decrease in L-FABP protein (*P*<0.001) and mRNA (*P*<0.05), as well as increased poly-ubiquitinated L-FABP (*P*<0.001). Sites of 4-HNE adduction on mouse recombinant L-FABP were mapped using MALDI-TOF/TOF mass spectrometry on apo (Lys57 and Cys69) and holo (Lys6, Lys31, His43, Lys46, Lys57 and Cys69) L-FABP. The impact of 4-HNE adduction was found to occur in a concentration-dependent manner; affinity for the fluorescent ligand, anilinonaphthalene-8-sulfonic acid, was reduced from 0.347 µM to Kd_1_ = 0.395 µM and Kd_2_ = 34.20 µM. Saturation analyses revealed that capacity for ligand is reduced by approximately 50% when adducted by 4-HNE. Thermal stability curves of apo L-FABP was also found to be significantly affected by 4-HNE adduction (ΔTm = 5.44°C, P<0.01). Computational-based molecular modeling simulations of adducted protein revealed minor conformational changes in global protein structure of apo and holo L-FABP while more apparent differences were observed within the internal binding pocket, revealing reduced area and structural integrity. New solvent accessible portals on the periphery of the protein were observed following 4-HNE modification in both the apo and holo state, suggesting an adaptive response to carbonylation. The results from this study detail the dynamic process associated with L-FABP modification by 4-HNE and provide insight as to how alterations in structural integrity and ligand binding may a contributing factor in the pathogenesis of ALD.

## Introduction

Chronic ethanol consumption is a prominent cause of liver disease and is responsible for significant morbidity and mortality throughout the Western world [1], [2], [3]. The pathogenesis of alcoholic liver disease (ALD) is progressive, complex, and highly variable resulting in a range of histologic abnormalities including steatosis and steatohepatitis. Disease progression is largely regarded to be a result of the oxidative metabolism of ethanol leading to an imbalance between pro-oxidant and antioxidant mechanisms [4], [5]. Previous reports have indicated initiation of cellular dysfunction from increased reactive oxygen species (ROS) by modifying and inactivating proteins, lipids and membranes, and DNA [6]. The generation of ROS has also been shown to result in the initiation of lipid peroxidation (LPO), whereby reactive aldehydes are generated from the oxidation of polyunsaturated fatty acids. Among the numerous aldehydes generated from LPO is the α,β-unsaturated aldehyde, 4-hydroxy-2-nonenal (4-HNE). This biogenic aldehyde is present under basal conditions and can adduct nucleophilic protein side-chains, namely Cys >> His > Lys residues through either Michael addition (MA) or Schiff base (SB) reactions [6], [7], [8]. It has been estimated that up to 95% of 4-HNE MAs are stabilized in the form of cyclic hemi-acetals (HA) [9], [10], [11]. The half-life of 4-HNE has been reported to be ∼2 minutes; demonstrating that it can migrate from the site of origin to other intracellular sites [7]. High levels of 4-HNE protein adducts have been found in the livers of patients with hemochromatosis, Wilson’s disease, primary biliary cirrhosis, advanced stages of ALD and in the serum of type 2 diabetes patients [12], [13]. Given its high reactivity and ability to covalently modify proteins, it is important to understand the consequence of protein adduction by 4-HNE and how these reactions may contribute to the pathogenesis and/or progression of numerous human disease states.

Liver fatty acid-binding protein (L-FABP) coordinates lipid responses in the cell by facilitating the cytosolic transport of fatty acids (FA) to various compartments, including lipid droplets, the endoplasmic reticulum, mitochondrion, peroxisomes, and the nucleus [14]. L-FABP is comprised of ten antiparallel β-sheets (βA- βJ) encapsulating a solvent-accessible ligand-binding pocket, capped by an N-terminal helix-turn-helix motif (αI-αII) [15]. It is a unique member of the intracellular lipid-binding protein family in that it can stoichiometrically bind two hydrophobic ligands, with the highest affinity for long chain fatty acids (LCFA) [15], [16], [17]. These C12-C20 LCFA ligands are the substrates that perpetuate the cycling of lipid peroxidation. The localization and function of L-FABP directly places it within the microenvironment containing reactive aldehydes; therefore, susceptibility to adduction by reactive aldehydes remains high. Due to its high abundance in the cell, accounting for up to 5% of total cytosolic protein in hepatocytes, L-FABP is proposed to play an important role in hepatic lipid homeostasis. Alterations in protein stability and activity from aldehyde adduction may contribute to the changes in lipid metabolism that are observed in disorders associated with hepatic lipid accumulation such as ALD.

The expression of L-FABP is highly affected following ethanol consumption, as demonstrated by an approximate 30% decrease in rats [18]. This report also revealed immunopositive staining for hepatic 4-HNE adducted L-FABP. The mechanisms for the observed decrease in protein expression remain unknown; however, it is possible that 4-HNE adduction results in a destabilization of L-FABP, targeting the protein for degradation. In the present study, we report a significant decrease in L-FABP protein and FABP1 mRNA, as well as an increase in ubiquitintated L-FABP following ethanol consumption in mice. We have identified sites of 4-HNE adduction on both apo (unbound) and holo (lipid-bound) L-FABP and have investigated the consequences of this modification on protein function, stability, and structure. The impact of 4-HNE modification resulted in diminished ligand binding, altered protein stability, and dramatic changes around the ligand accessible portals of the protein. Data are also provided demonstrating a significant impact of 4-HNE adduction on L-FABP structural and functional dynamics that may be contributing factors for altered lipid homeostasis in ALD.

## Materials and Methods

### Reagents

Unless otherwise specified, all reagents were purchased from Sigma Aldrich (St. Louis, MO).

### Ethanol Feeding Model

All protocols were approved by the Institutional Animal Care and Use Committee (IACUC) at the University of Colorado Anschutz Medical Campus. Male C57Bl/6J mice were fed a modified Lieber-DeCarli Diet (BioServ, Frenchtown, NJ) consisting of 45% fat-derived calories and 15% protein-derived calories with the remaining caloric content derived from either ethanol or maltose-dextrin. Mice were fed for a period of 6 weeks according to previous laboratory studies [19], [20]. At termination, the liver was harvested for whole tissue and histological evaluation as previously described [20], [21]. Subcellular fractions were isolated from whole liver tissue using sucrose gradient centrifugation [22].

### 2-Dimensional Gel Electrophoresis

Enriched cytosolic fractions were assessed utilizing 2D separation techniques as previously described [21], [23]. Briefly, 250 µg of cytosolic protein was separated utilizing 11 cm IPG strips with a pH gradient of 3–10 in a Protean IEF cell (Biorad Lifesciences, Hercules, CA). Separation in the second dimension was performed utilizing 11 cm 8–16% SDS-PAGE gradient gels (Biorad Lifesciences). Protein gels were either stained with Imperial protein stain (Thermofisher, Rockford, IL) or transferred to Polyvinylidene fluoride (PVDF) and blocked with 5% non-fat dry milk (NFDM) (w/v) in tris-buffered saline with 0.2% tween-20 for 30 min at RT. The membranes were probed using a custom primary antibody directed against 4-HNE modified proteins (Bethyl Laboratories, Montgomery, TX) or L-FABP (Abcam, Cambridge, MA) utilizing standard Western blotting techniques. Membranes were developed with enhanced chemiluminescence and scanned using a STORM 860 Scanner (Molecular Dynamics, Sunnyvale, CA). Developed Western blots were overlaid to stained protein gels and immunopositive spots were carefully matched, excised and digested with trypsin. Peptides were subsequently analyzed utilizing an 1100 Series LC/MSD Trap (Agilent Technologies, Santa Clara, CA). Protein identifications were made utilizing data analysis software MASCOT, with protein identifications above 54 being significant hits.

### Co-immunoprecipitation of Ubiquitinated L-FABP

200 ug of whole cell homogenates from both control and ethanol mice (n = 6/group) were incubated with 5 ug of mouse monoclonal ubiquitin antibody (Abcam, cat# ab411) for 2 hours at 4°C on a rotator to allow ubiquitin protein to bind to antibody. 100 ul of a 50% Protein G slurry (prepared and stored in 0.02% sodium azide and PBS, pH 7.4) was added to the reaction and incubated at 4°C rotating overnight. Samples were washed 5×5 minutes in PBST (PBS +2% Tween20) and denatured in SDS-PAGE loading buffer at 95°C for 5 minutes. Immunoprecipitated protein was separated via SDS-PAGE and transferred to PVDF, and immunoblotted for L-FABP (Abcam, cat # 7847). Density of ubiquitinated bands were normalized to levels of L-FABP/β-Actin determined by immunoblotting.

### RT-PCR Analysis of FABP1

Total RNA was isolated from 20 ug of frozen tissue from control and ethanol-fed mice (n = 3) using RNeasy Mini Kits (Qiagen,Valencia, CA). 1 ug of RNA was reverse-transcribed to cDNA at 42°C for 60 minutes using AMV reverse-transcriptase (New England Biolabs, Ipswich, MA) with oligo(dT) Primers (Invitrogen, Carlsbad, CA). cDNA was amplified using GoTaq qPCR Master Mix (Promega, Madison, WI) and gene specific primers for FABP1 (F: 5′-GCA GAG CCA GGA GAA CTT TG-3′; R: 5′-GGG TCC ATA GGT GAT GGT GAG-3′ [24] and G3PDH (F: 5′-GGT GTG AAC GGA TTT GGC CGT ATT-3′; R: 5′-TGG AAG AGT GGG AGT TGC TGT TGA-3′ [25] (IDT, Coralville, IA).

### Cloning, Expression and Purification of L-FABP

The L-FABP (*FABP1*) gene from *mus musculus* was TOPO cloned into pET 100 vector (Invitrogen) from the pCMV-SPORT6 vector containing clone #4159971 (Open Biosystems, Huntsville, AL). All constructs were verified by sequence at the University of Colorado Cancer Center, and transformed into BL21 (DE3) *Escherichia coli*. Cultures were grown overnight with ampicillin selection, and used to propagate a larger 1 L culture until OD_600_ reached 0.6–0.8. Cultures were then induced with 1 mM IPTG for 4 hours, sedimented via centrifugation at 6,000× g, flash frozen and stored at −80°C until purification. The bacterial pellet was lysed in 50 mM tricine (pH 8.0), 500 mM NaCl, 1 mM β-mercaptoethanol (BME), 1 mM EDTA, protease inhibitor cocktail and lysozyme and sonicated using a Branson Digital Sonifier (Danbury, CT). Following centrifugation, the supernatant containing recombinant L-FABP (rL-FABP) was purified initially using ammonium sulfate precipitation up to 65% saturation, dialyzed overnight with three subsequent buffer changes of 50 mM Tricine (pH 8.0), and concentrated using an amicon ultrafiltration stirred cell (Millipore, Billerica, MA). The protein sample was further purified using an ÄKTA FPLC (GE Healthcare, Piscataway, NJ) with anion exchange chromatography (HiPrep QX L 16/10). Purity of fractions were analyzed via SDS-PAGE and Coomassie Blue staining and the final rL-FABP prep was determined to be >95% purity. rL-FABP was delipidated using established protocols [26], [27]. The purified, delipidated rL-FABP was stored in 1 mg/ml aliquots containing 10% sucrose at −80°C until required. For the holo (lipid-bound) isoform of rL-FABP, protein was incubated with 1 mM linoleic acid (LA) (from a 10 mM stock solubilized in 100% ethanol) for 1 hour at 25°C rotating end-over-end say followed by dialysis in tricine buffer (pH 8.0) overnight at 4°C. Bound lipid was verified using an established fluorescence assay [28], [29].

### Modification of rL-FABP with 4-HNE

rL-FABP was incubated with increasing molar ratios of 4-HNE (0.5X, 1X, 5X, and 10X [5.85 µM, 11.7 µM, 58.52 µM, and 117.03 µM]) for 1 hour at 25°C in PBS (pH 7.4). Following modification, samples were then reduced with 10 mM NaBH_4_ for 30 minutes at 25°C to trap transient adducts. Aldehyde-modified rL-FABP protein samples were separated on a polyacrylamide gel via standard SDS-PAGE procedures and stained with Coomassie Blue.

### Mass Spectrometry Analysis of rL-FABP

5 µg of 4-HNE treated r-LFABP (reduced and non-reduced) was separated via SDS-PAGE and stained with Coomassie blue. Bands were then excised, digested with sequencing-grade trypsin (Promega, Madison, WI), purified using C-18 ziptips (Millipore) and then spotted 1∶1 with α-cyano-4-hydroxycinnamic acid matrix onto an Opti-TOF 96-well insert (Applied Biosystems, Carlsbad, California) for analysis. Peptides were analyzed on an ABI 4800 Plus Matrix-assisted laser desorption ionization time-of-flight (MALDI-TOF/TOF) mass spectrometer using Reflector acquisition mode, potential 4-HNE adducts were then identified using Mascot (v 2.1.04, www.matrixcience.com). Collision-induced dissociation fragmentation was then utilized to acquire b/y ion fragmentation spectra for adducted peptides, which were aligned with predicted ion fragmentation pattern using ProteinProspector v 5.4 (http://prospector.ucsf.edu, USCF Mass Spectrometry Facility).

### rL-FABP Binding and Displacement Using 1-Anilinonaphthalene-8-Sulfonic Acid (ANS)

Steady-state fluorescence spectra were measured using a HORIBA JOBIN YVON FluoroMax-3 Fluorometer (Edison, NJ USA). The binding of ANS to rL-FABP was monitored following excitation at 370 nm and emission at 475 nm, with slits set at 2 and 4 nm, respectively. Measurements were taken at ambient temperature in a 0.7 mL fluorescence cuvette (Starna Cells, Atascadero, CA). Equilibrium binding studies were conducted with saturating concentrations of ANS according to previous studies [29], [30] with slight modifications. Briefly, ANS (0–45 µM) was titrated into 1 µM rL-FABP in 50 mM tricine buffer (pH 8.0), mixed for 30 seconds, and emission spectra were recorded. rL-FABP was either treated with 4-HNE (0.1X [5.93 µM], 1X [53.93 µM], or 5X [245.09 µM]) or the arginine blocker phenylglyoxal (PG) (10 mM) [31], or a combination of the two (PG +5X). Following treatments, protein was dialyzed overnight in tricine buffer, pH 8.0. Preparation of ANS was conducted according to Denicola et al. [29], where it was solubilized in absolute ethanol and quantified at Abs_372_ with an extinction coefficient of 8,000 cm^−1^·M^−1^ prior to use.

Lipid displacement assays were adapted from previous publications [28], [30]. Briefly, lipids were used to displace ANS bound to rL-FABP to assess how 4-HNE modification affects affinity towards more biologic ligands. Lipid stocks were diluted in absolute ethanol (10 mM, 5 mM, 1 mM, 0.1 mM) and prepared in a nitrogen gas chamber. Using conditions similar to ANS binding, increasing concentrations of FAs [stearate (SA), oleate (OA), and LA] were titrated into a pre-equilibrated solution containing 45 µM ANS, 1 µM r-LFABP, and 50 mM tricine (pH 8.0). The sample was mixed for an additional 30 seconds and the excitation and emission of ANS was measured as mentioned, where the loss of fluorescence indicates affinity towards the lipid.

### Thermal Denaturation of rL-FABP

The stability of rL-FABP was assessed using UV-monitored heating experiments. Native (apo and holo) and 4-HNE modified (apo and holo) rL-FABP (0.8 mg/ml) was thermally denatured in 50 mM tricine buffer (pH 8.0) at 40–95°C over 1.5 hours in a 0.7 mL quartz cell (Starna Cells). Absorbance of rL-FABP was monitored at 260–360 nm using a Chirascan Plus Spectrometer (Applied Photophysics, Leatherhead, Surrey, UK) once per minute. Temperature was continuously ramped 1°C/min. Light scattering was recorded continuously at 360 nm to monitor protein aggregation [32]. Temperature of aggregation (Tm) was determined using Global Analysis T-Ramp software provided by Applied Photophysics (Pro-Data Global3 v1.1.0) following baseline correction.

### Molecular Modeling

All simulations were performed using Discovery Studio software (v3; Accelrys Inc., San Diego, CA). The crystallographic coordinates of the 2.3Å rat FABP1 crystal structure (PDB: 3GLS) [33] were obtained from the RCSB Protein Data Bank (http://wwww.rcsb.org) and used to generate a homology model of the mouse FABP1 crystal (PDB 1LFO, 98% sequence identity) [33]. The sequences of the proteins were aligned and five homology models were created and structural evaluation was performed on the basis of discrete optimized protein energy score [34]. The most energy favored intermediate was retained and the residues were corrected for physiological pH. The homology model was refined using CHARMm and subjected energy minimization (steepest descent, 100 iterations; conjugate gradient, 1000 iterations) to a convergence of 0.001 kcal/mol using the Generalized Born implicit solvent model. The minimization of the native apo L-FABP resulted in an overall potential energy of 3993.5 kcal/mol. Michael adducts of 4-HNE were built onto the Cys69 and Lys57 residues and the model was subject to a further round of minimization. LA was docked into the binding pocket of the unadducted FABP1 homology model using the flexible docking protocol [35],and the Generalized Born method was used to calculate binding energies (ΔG_bind_) [36], with a non-bonded cut-off radius of 12Å. The LA molecules adopted similar conformations as OA, which has been previously co-crystalized with rat L-FABP [33]. The ΔG_bind_ for LA at the base of the FABP1 binding pocket was −35.6 kcal/mol and −35.7 kcal/mol at the entrance of the binding pocket. The total potential energy post-minimization of native holo L-FABP was 7983.2 kcal/mol. Michael addition and Schiff base 4-HNE adducts were then built onto Cys69, His43, Lys31, Lys57, Lys6 and Lys46 residues and the complex was subjected to energy minimization. Cyclic HA 4-HNE adducts were also built in place of the MA adducts on apo and holo L-FABP and subjected to energy minimization.

### Data Analysis

Statistical analysis and generation of graphs was performed using GraphPad Prism (v 4.02; GraphPad Software, San Diego, CA) and SigmaPlot (v10.0; Systat Software, San Jose, CA). Statistical differences between control and treated samples were assessed using a paired Student’s *t* test or a one-way ANOVA with Tukey’s post-test. Differences were considered significant if *p*<0.05. Nonlinear regression (curve fit) analysis was performed using a two-site binding hyperbola (eq 1), where ΔF is the enhancement in fluorescence intensity upon the binding of ANS to L-FABP to a point of saturation (B_max_).
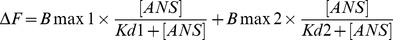
(1)


For the displacement assays, one-site inhibition analysis of the data was utilized based on the fit of the data (instead of a two-site inhibition analysis) (eq2). Fluorescence counts that are inhibitable are denoted as b_i_, and residual counts are b_r_. The concentration of OA, SA or LA is denoted as [FA]. The Hill constant, η, indicates the cooperativity between the binding sites, where if η >1 the sites are positively cooperative, η <1 the site are negatively cooperative, and if η  =  1 the sites are non-cooperative.
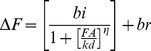
(2)


## Results

### Hepatic L-FABP is a Target for Modification by 4-HNE in Ethanol-fed Mice

**Figure 1:**
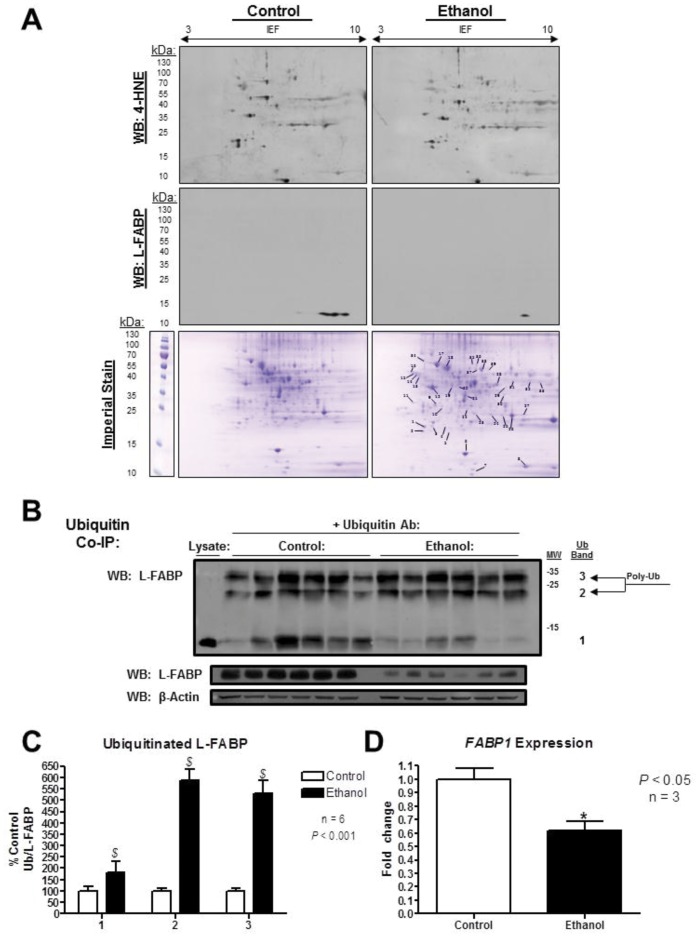
L-FABP was identified as a 4-HNE modified protein in cytosolic fractions of mice chronically treated with ethanol utilizing 2D SDS-PAGE and LC-MS. (A) 48 proteins in 39 spots were identified from 2D gels by matching up immunoblots probed with an anti-4-HNE antibody created in our lab. Immunoblots from both control and ethanol-fed mice reveal an increase in the total pool of 4-HNE modified proteins. A second set of gels were probed for L-FABP, while a third set was stained with imperial and used for protein excision, tryptic digest, and LC-MS identification. (B) *In vivo* stability of L-FABP was assessed via measurement of ubiquitin-tagged protein by co-immunoprecipitation with ubiquitin antibody. (C) Relative quantification of poly-ubiquitinated L-FABP was normalized to total L-FABP in the liver (*P*<0.001, n = 6). (D) FABP1 mRNA was measured by qPCR (*P*<0.05, n = 3).

LPO is a known consequence to sustained ethanol consumption, and identifying protein targets for modification by reactive aldehydes may further elucidate mechanistic information for the progression of specific diseases. Mice fed an ethanol-containing diet for 6 weeks has been shown to result in significant hepatic alterations consistent with early liver injury, including increased plasma ALT activity, increased liver triglyceride accumulation, and hepatomegaly [19], [20]. As shown in Figure 1A, the 2D gel immunostained for 4-HNE protein adducts of the ethanol-fed mice displayed a marked increase in 4-HNE-modified proteins. We have identified 48 unique proteins from 39 4-HNE immunopositive spots from hepatic cytoplasmic fractions of mice fed chronically with ethanol for 6 weeks. Of the 39 spots, 34 received significant protein identifications (Table S1). Interestingly, 4-HNE immunopositive spots are present in the control gels, but to a lesser degree as compared with the ethanol group. The presence of 4-HNE in the control is expected as submicromolar to micromolar concentrations of 4-HNE have been shown to induce homeostatic, cell-specific effects such as stimulation of proliferation and regulating signaling pathways [37]. Of the proteins identified, our focus remained on L-FABP due to its pivotal role in hepatic FA uptake and trafficking. Following tryptic digest, 4 peptides of L-FABP were identified from the 2D gels, with 34% sequence coverage and a significant MASCOT score of 303. To verify spot identification, paired gels were probed with an antibody directed against L-FABP. Compared with the control gel, the ethanol-fed sample showed a reduction in the total expression of L-FABP; correlating with previous data observed in rats [18]. In Figure 1B, we confirm the ∼80% reduction in L-FABP by 1D Western Blot analysis in whole cell lysates of control and ethanol-fed mice (*P*<0.001, n = 6). In Figure 1B and 1C the stability of L-FABP was assessed *in vivo.* Co-immunoprecipitation of ubiquitinated L-FABP revealed a significant increase in poly-ubiquitinated L-FABP in response to ethanol treatment when normalized to the total L-FABP protein pool (*P*<0.001, n = 6). Figure 1D shows RT-PCR of FABP1 mRNA, which revealed a 1.6 fold decrease in message following ethanol treatment. Relative expression of FABP1 in control (1.00±0.084) and ethanol (0.617±0.069) livers (*P*<0.05, n = 3).

### Identification of 4-HNE Adducts on Both Apo and Holo rL-FABP

**Figure 2:**
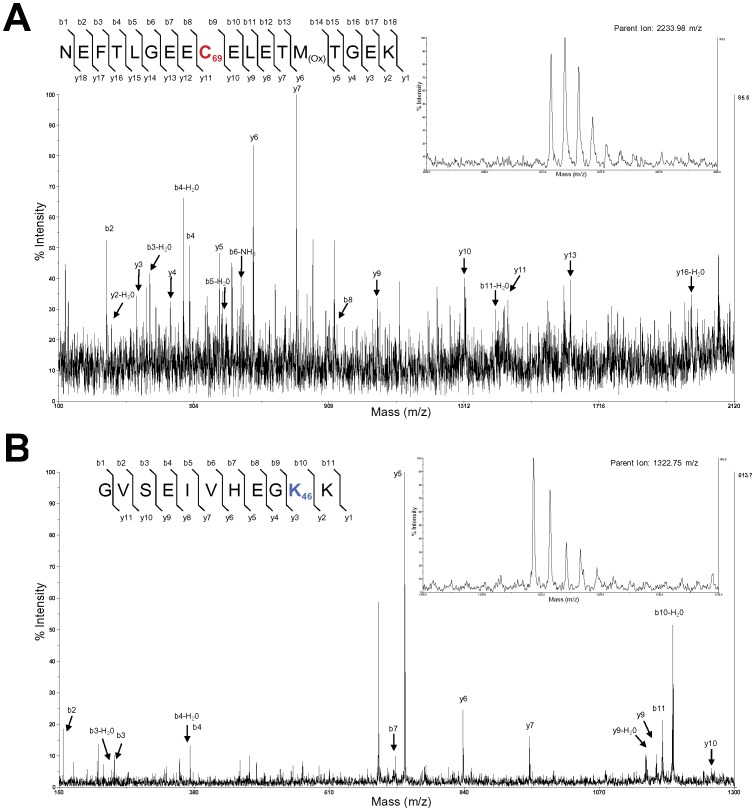
4-HNE adducts were identified on apo and holo rL-FABP via MALDI-TOF/TOF Mass Spectrometery. Following incubation with reactive aldehyde, rL-FABP was found to be modified in both the apo and holo form. MS/MS spectra from 4-HNE treated apo rL-FABP revealed the presence of two protein adducts, at residues Lys57 and Cys69. The same experiments were conducted with holo rL-FABP and revealed an additional four 4-HNE adducts on Lys31, Lys46, Lys6, and His 43. Identified b/y ions are labeled within the spectrum and shown along the peptide backbone above the spectrum. The parent ion of the MS/MS fragment is shown as an inset in the upper right corner of the spectrum.

L-FABP exhibits a high affinity for a vast range of hydrophobic ligands, including LCFA [38]. Structurally, L-FABP changes conformation depending on the apo or holo state [39]; it was therefore hypothesized that 4-HNE adduction would differ based on the binding state of the protein. Based on the complications of identifying *in vivo* modifications, adduction of rL-FABP by 4-HNE was performed *in vitro* to identify specific sites of modification in apo and linoleate-bound protein. As demonstrated in Table 1, MALDI-TOF/TOF analysis of digested rL-FABP revealed two MA 4-HNE adducts on apo rL-FABP, at Lys57 and Cys69 with sequence coverage of 89% and 87%, respectfully. The holo form revealed six total adducts with 90% sequence coverage. These were identified at Lys31, His43, Lys57 and Cys69 MA and Lys6 and Lys46 SB adducts. Figure 2A and 2B display the MS/MS spectrum of parent ions containing Cys69 of apo rL-FABP and Lys46 of holo rL-FABP, where relative y and b ions confirm all 4-HNE adducts on rL-FABP. The remaining MS/MS spectra of the 4-HNE adducted peptides also confirmed modifications on Lys57, His43, Lys6, and Lys31 of rL-FABP (Figures S1 and S2).

**Table 1:** rL-FABP peptides identified with 4-HNE Michael addition and Schiff bases adducts.

	Residues	Mass	Miss	Sequence	Peptide	Type	Mass Shift	Molar Excess 4-HNE	4-HNE [µM]
***Apo rL-FABP***	*61–78*	2215.98	0	NEFTLGEECELETMTGEK	**Cys69**	*MA*	156	10X*	117.03
	*61–78*	2231.98	0	NEFTLGEECELETM(ox)TGEK	**Cys69**	*MA*	156	10X*	117.03
***Holo rL-FABP***	*61–78*	2231.94	0	NEFTLGEECELETM(ox)TGEK	**Cys69**	*MA*	156	10X*	117.03
***Apo rL-FABP Reduced***	*50–60*	1404.87	1	LTITYGPKVVR	**Lys57**	*MA*	158	0.5X*, 1X*, 5X*, 10X*	5.85, 11.7, 58.52, 117.03
***Holo rL-FABP Reduced***	*61–78*	2233.98	0	NEFTLGEECELETM(ox)TGEK	**Cys69**	*MA*	156	1X*, 5X*, 10X*	11.7, 58.52, 117.03
	*50–60*	1404.87	1	LTITYGPKVVR	**Lys57**	*MA*	158	10X*	117.03
	*37–46*	1212.65	0	GVSEIVHEGK	**His43**	*MA*	158	0.5X, 1X*, 5X*, 10X*	5.85, 11.7, 58.52, 117.03
	*21–33*	1539.89	1	AIGLPEDLIQKGK	**Lys31**	*MA*	158	5X*, 10X*	58.52, 117.03
	*19-Jan*	2609.17	1	M(ox)NFSGKYLQSQENFEPFMK	**Lys6**	*SB*	140	10X*	117.03
	*19-Jan*	2625.16	1	M(ox)NFSGKYLQSQENFEPFM(ox)K	**Lys6**	*SB*	140	1X, 5X, 10X*	11.7, 58.52, 117.03
	*50–60*	1386.85	1	LTITYGPKVVR	**Lys57**	*SB*	140	5X, 10X*	58.52, 117.03
	*48–60*	1628.03	2	IKLTITYGPKVVR	**Lys57**	*SB*	140	5X, 10X*	58.52, 117.03
	*37–47*	1322.75	1	GVSEIVHEGKK	**Lys46**	*SB*	140	5X, 10X*	58.52, 117.03

* indicates conformation of protein adduct by MS/MS.

Type of adduct refers to either (1) MA  =  Michael addition or (2) SB  =  Schiff base.

Modifications were identified on Cys69 of both apo and holo rLFABP. Following reductive stabilization with sodium borohydride in both apo and holo rL-FABP, transient adducts were identified at His43, Lys6, Lys31, Lys57, and Lys46.

### 4-HNE Adduction Alters the Capacity and Affinity for ANS

**Figure 3:**
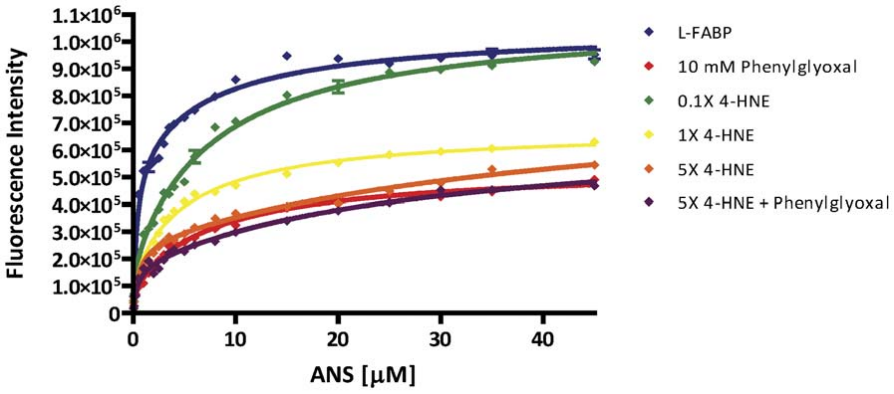
The binding profile of apo rL-FABP is adversely affected following 4-HNE adduction. Utilizing the fluorescent probe, ANS, equilibrium binding studies of native and 4-HNE modified r-LFABP revealed alterations in both affinity for ANS and maximal binding capacity for ligands when the protein is modified. PG, an arginine blocker, is used as a control for 50% of maximal binding. Blue - 0X 4-HNE; green-0.1X 4-HNE (5.93 µM); yellow-1X 4-HNE (53.95 µM); orange-5X 4-HNE (245.09 µM); red-PG (10 mM); purple-5X 4-HNE + PG.

To further assess the functional effects of 4-HNE adduction on L-FABP, a well-established binding assay utilizing ANS was performed. As shown in Figure 3, a significant, concentration-dependent reduction in the capacity and affinity for ANS was observed following adduction with pathologically relevant concentrations of 4-HNE. Table 2 contains the regression analysis comparing the native and 4-HNE adducted rL-FABP. The dissociation constants in the primary binding site show increased affinity for ANS at lower concentrations of 4-HNE (0.1X, kd_1_ = 0.132±0.074 µM and 1X, 0.135±0.074 µM compared to native (0X, 0.347±0.153 µM ) but relatively minor changes at the highest concentration assessed (5X, kd_1_ = 0.395±0.101 µM ). The secondary binding site, however, displayed an approximate 6-fold decrease in the affinity for ANS following 4-HNE modification at the highest 4-HNE treatment (0x, kd_2_ = 6.17±1.44 µM compared to 5X, kd_2_ = 34.20±13.85 µM). The Bmax_1_ values of 4-HNE modified rL-FABP compared to the native protein demonstrate reduced capacity in the primary site following 4-HNE modification in a concentration dependent manner. The Bmax_1_ is reduced the most by PG (83, 200±23,200 AFU) compared to native (540,200±142,500 AFU), consistent to previously published reports [31]. We also observed that Bmax_2_ values remain relatively unchanged following 4-HNE adduction.

**Table 2:** Equilibrium binding data utilizing the fluorescent probe ANS.

	L-FABP	PG	0.1X	1X	5X	5X+PG
***Bmax1 (AFU)***	540,200±142,500	83,200±23,200	175,500±36,800	123,000±28,900	238,100±21,700	168,200±22,900
***Kd_1_ (µM)***	0.347±0.153	0.165±0.111	0.132±0.074	0.135±0.074	0.395±0.101	0.326±0.127
***Bmax2 (AFU)***	500,500±116,800	446,400±17,500	903, 400±28,300	548,900±23,700	537,400±81,900	512,200±64,500
***Kd_2_ (µM)***	6.17±1.44	7.18±0.121	7.40±1.07	4.99±0.71	34.20±13.85	27.89±10.71

Values presented are mean ± SEM of two independent experiments consisting of at least 3 replicates. AFU denotes arbitrary fluorescent units.

### 4-HNE Modification Alters the Binding Capacity and Affinity for Natural Ligands

**Figure 4:**
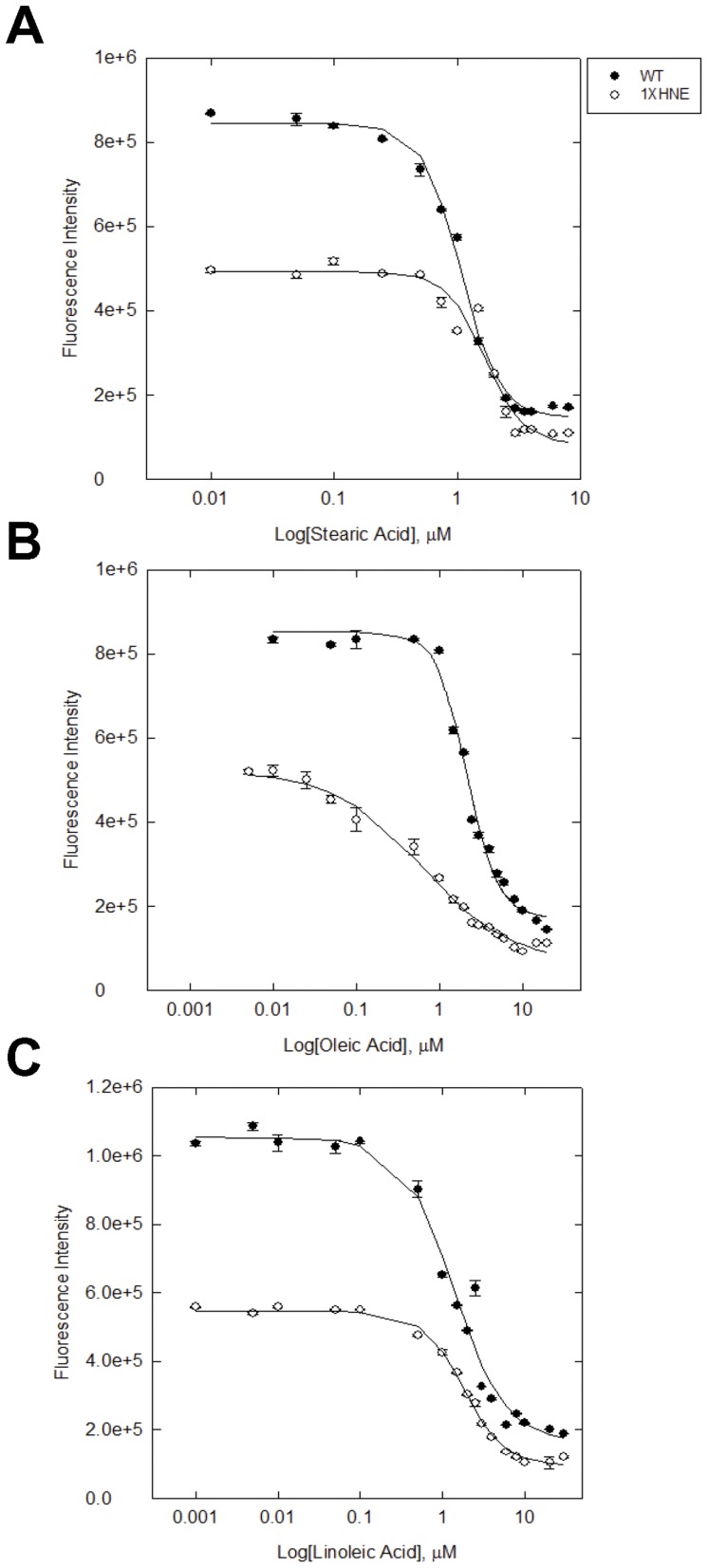
Affinity and total binding capacity for fatty acids is diminished following 4-HNE modification. The displacement of ANS by natural ligands of L-FABP is shown in both native and 1X 4-HNE (53.95 µM) treated rL-FABP. Displacement profiles of rL-FABP reveal loss of both the affinity and the total capacity for SA (A), OA (B), and LA (C).

The effects of 4-HNE modification on the affinity and capacity of L-FABP towards natural ligands, including FA, were analyzed using a well-documented ANS displacement assay. As demonstrated in Figure 4 and Table 3, 4-HNE reduces the maximal binding to the FA tested by 41% for SA, 32.7% for OA, and 49% for LA, respectively. The inhibition constants of adducted rL-FABP increased from the native protein for SA and LA, but decreased for OA. These data indicate that 4-HNE modification may affect the specificity towards ligands despite showing a decrease in the overall capacity of the system. The Hill constants indicate positive cooperativity for WT and 4-HNE adducted rL-FABP; with negative cooperativity for 4-HNE adducted rL-FABP displaced with OA.

**Table 3:** Displacement of ANS by stearic, oleic, and linoleic fatty acids.

		WT				1XHNE			
*FA*		b_i_ (AFU)	k_i_ (µM)	b_r_ (AFU)	n	b_i_ (AFU)	k_i_ (µM)	b_r_ (AFU)	n
***Stearic Acid***		698,800±19,400	1.19±0.13	145,800±14,400	2.75±0.264	411,800±36,800 ***^#^***	4.41±1.66	81,300±30,500	2.74±0.37
***Oleic Acid***		680,200±26,500000	5.88±1.45	171,600±19,800	2.31±0.29	457,600±26,400 ***^#^***	0.69±0.097	63,500±19,200	0.81±0.0999
***Linoleic Acid***		888,000±54	1.56±0.29	164,800±43,900	1.38±0.26	449,500±11,400 ***^#^***	2.80±0.30	96,300±9,200	1.76±0.14
									***^#^ P<0.01***

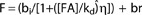
, where b_i_ denotes the counts inhibitable, k_i_ the inhibition constant, b_r_ the counts residual, *η* the hill coefficient and AFU the arbitrary fluorescent units. Values presented are mean ± SEM of duplicate independent experiments comprised of 3 or more replicates. All concentrations FA are µM.

### Stability Profiles of Native and 4-HNE Adducted L-FABP Differ in the Apo and Holo States

**Figure 5:**
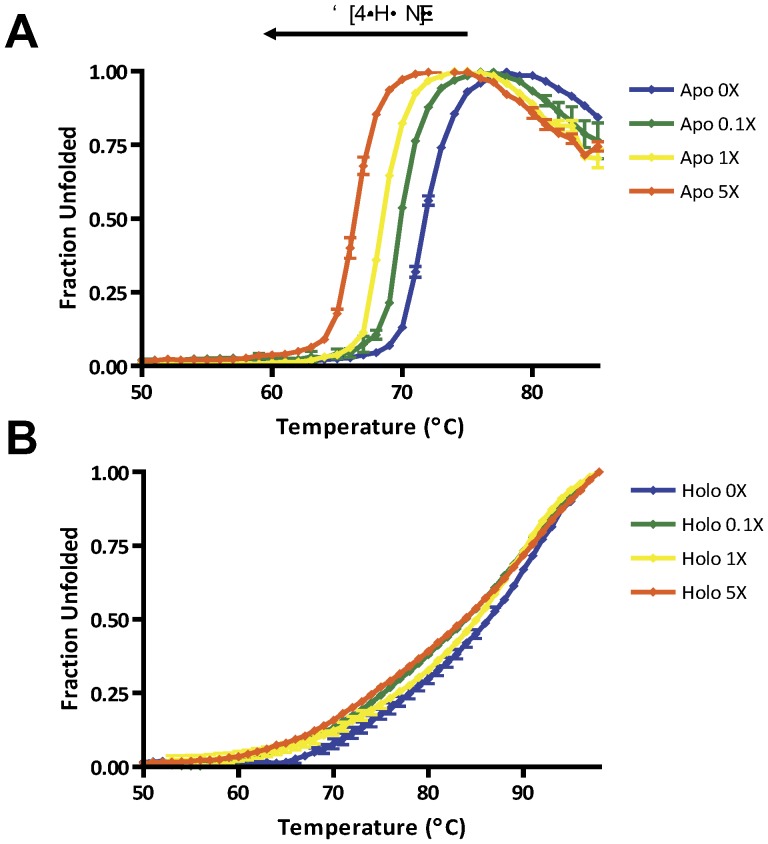
Adduction affects protein stability only when in the apo form. Thermal denaturation of apo and holo native and 4-HNE treated protein revealed differences in stability profiles. (A) The stability of apo rL-FABP decreases in a 4-HNE concentration dependent manner. (B) Holo rL-FABP thermal stabillity is only moderately affected by 4-HNE adducts. Blue - 0X 4-HNE (0 µM); green - 0.1X 4-HNE (4.93 µM); yellow - 1X 4-HNE (49.26 µM); orange - 5X 4-HNE (246.31 µM).

Consequences of 4-HNE modification commonly include protein inactivation and destabilization [40]. To further examine the effects of aldehyde adduction and destabilization of L-FABP, a thermal denaturation assay was employed. Figure 5A shows that apo rL-FABP (T_m_ = 71.82±0.09°C) is sensitive to degradation following 4-HNE modification in a concentration dependent manner (0.1X, T_m_ = 70.09±0.004°C; 1X, T_m_ = 68.56±0.05°C; 5X, T_m_ = 66.38±0.15°C; p<0.01). Contrary to apo rL-FABP, Figure 5B demonstrates that the thermal stability of holo rL-FABP (T_m_ = 86.09±0.57°C) is only moderately affected following 4-HNE adduction (0.1X, T_m_ = 83.84±0.39°C; 1X, T_m_ = 84.89±0.48°C; 5X, T_m_ = 83.698±0.53°C). Apo and holo stability may also be related by comparing the slope of the thermal melts, with the apo being far steeper than the holo indicating a faster transition between the folded and unfolded state. Consistent with previous reports, it is evident that lipid binding by FABP results in a significantly more stable product [41]. The T_m_ from the holo rL-FABP remains semi-quantitative as maximal temperature (98°C) does not result in a fully aggregated state. These data suggest that 4-HNE adduction only minimally affects holo rL-FABP protein stability.

### Molecular Modeling Reveals Slight Structural Changes Following 4-HNE Adduction

**Figure 6:**
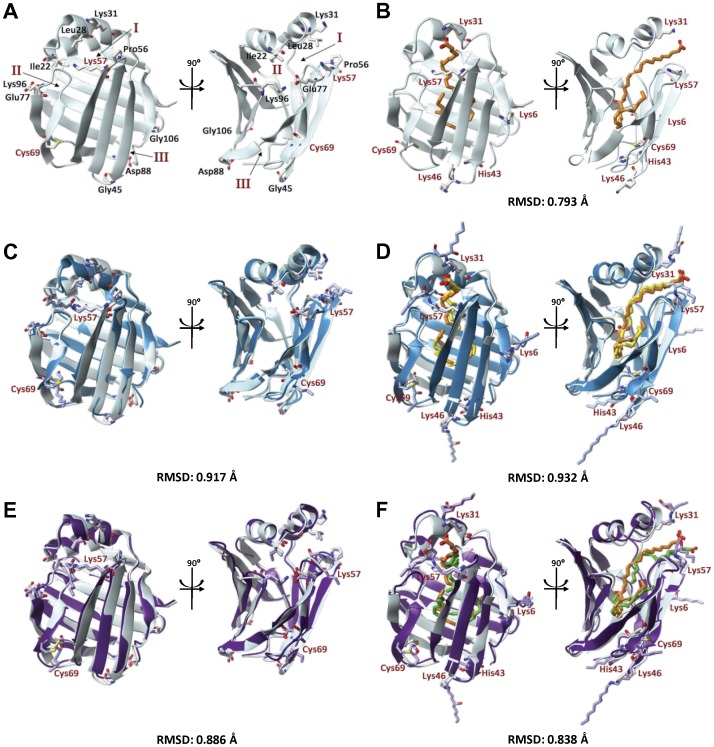
Molecular modeling simulations reveal minimal conformational changes in both apo and holo L-FABP as a result of peripheral 4-HNE adducts. Native apo (A) and holo (B) L-FABP is shown as the grey ribbon structure, “open chain” 4-HNE adducts and HA 4-HNE structures are shown as overlays over native protein, in blue (C and D) and purple (E and F), respectively. All structures are shown as a “front view” and 90° turned “side view”. Reference residues surrounding the three ligand portals are shown in black of the apo native structure and have been highlighted previously [62]. Root-mean-square deviation (RMSD) values were obtained comparing 4-HNE modified protein to the native. Residues found to be modified with 4-HNE are labeled in red, and are referenced in all protein structures. Docked LA is colored according to the 4-HNE adduction state: native  =  orange, 4-HNE  =  yellow, 4-HNE HA  =  green.

We have identified peripheral 4-HNE adducts on both the apo and holo rL-FABP utilizing MALDI-TOF/TOF studies. The structural consequence of these modifications was further investigated in the present study utilizing *in silico* simulations. Figure 6 demonstrates that the minimizations of these models resulted in minor structural differences between native, 4-HNE adducted, and the cyclized 4-HNE HA L-FABP in apo and holo states, respectively. Table 4 reveals the degree of structural shift between the alpha carbons (Cα) of portal 1 (Lys28, Lys31, Pro56), portal 2 (Ile22, Glu77, Lys96), and portal 3 (Ile48, Asp88, Gly105) in the 4-HNE adducted structures and the native protein. These indicate subtle structural changes occurring throughout the entire protein as a consequence of peripheral 4-HNE adduction, with the greatest shifts being under 3 Å.

**Table 4:** Distance of alpha carbons of critical reference residues surrounding the three ligand portals of L-FABP.

	Cα Shift in ReferenceResidues (Å)		Portal 1			Portal 2			Portal 3	
		28	31	56	22	77	96	48	88	106
***Apo L-FABP***	*4-HNE Adduct*	0.781	1.93	0.585	0.56	0.919	0.567	2.505	0.494	0.937
	*Hemiacetal*	0.62	0.303	0.784	0.508	0.976	0.844	2.046	0.24	0.864
***Holo L-FABP***	*4-HNE Adduct*	0.492	0.599	1.04	1.459	0.339	1.032	1.679	0.766	1.224
	*Hemiacetal*	1.242	0.904	0.395	1.689	0.201	0.947	0.684	0.577	1.236

These alpha carbons (Cα) of portals 1 (Lys28, Lys31, Pro56), 2 (Ile22, Glu77, Lys96), and 3(Ile48, Asp88, Gly105) in the 4-HNE adducted structures were compared to those of the native protein to identify structural shifts.

Utilizing PROPKA (http://propka.ki.ku.dk/), the predicted pKa and solvent accessibility of the six 4-HNE susceptible residues were calculated in the native protein. Modified residues in apo and holo-L-FABP are variable for all residue types, indicating that there is a range of “optimal” factors for 4-HNE to react with protein side-chains. More specifically, it appears the nucleophilic softness and chemical potential are more important factors than pH to regulate the nucleophilicity of an amino acid [42]. In the apo state, Lys57 and Cys69 are 21% and 11% buried from the solvent, with a pKa value of 10.22 and 10.07, respectively. In the holo state, Cys69 and His43 are both 37% buried with a pKa of 9.57 and 5.23. Lys6, Lys 31, Lys46, and Lys57 in the holo state are more solvent accessible (4%, 0%, 0% and 28% buried) with similar pka values (10.97, 12.33, 10.49 and 9.61). The “percent buried” factor of the residue reveals that solvent accessibility remains highly important for 4-HNE adduction to occur i.e. the most buried residues are Cys69 and His43 on holo L-FABP at 37% each.

**Figure 7:**
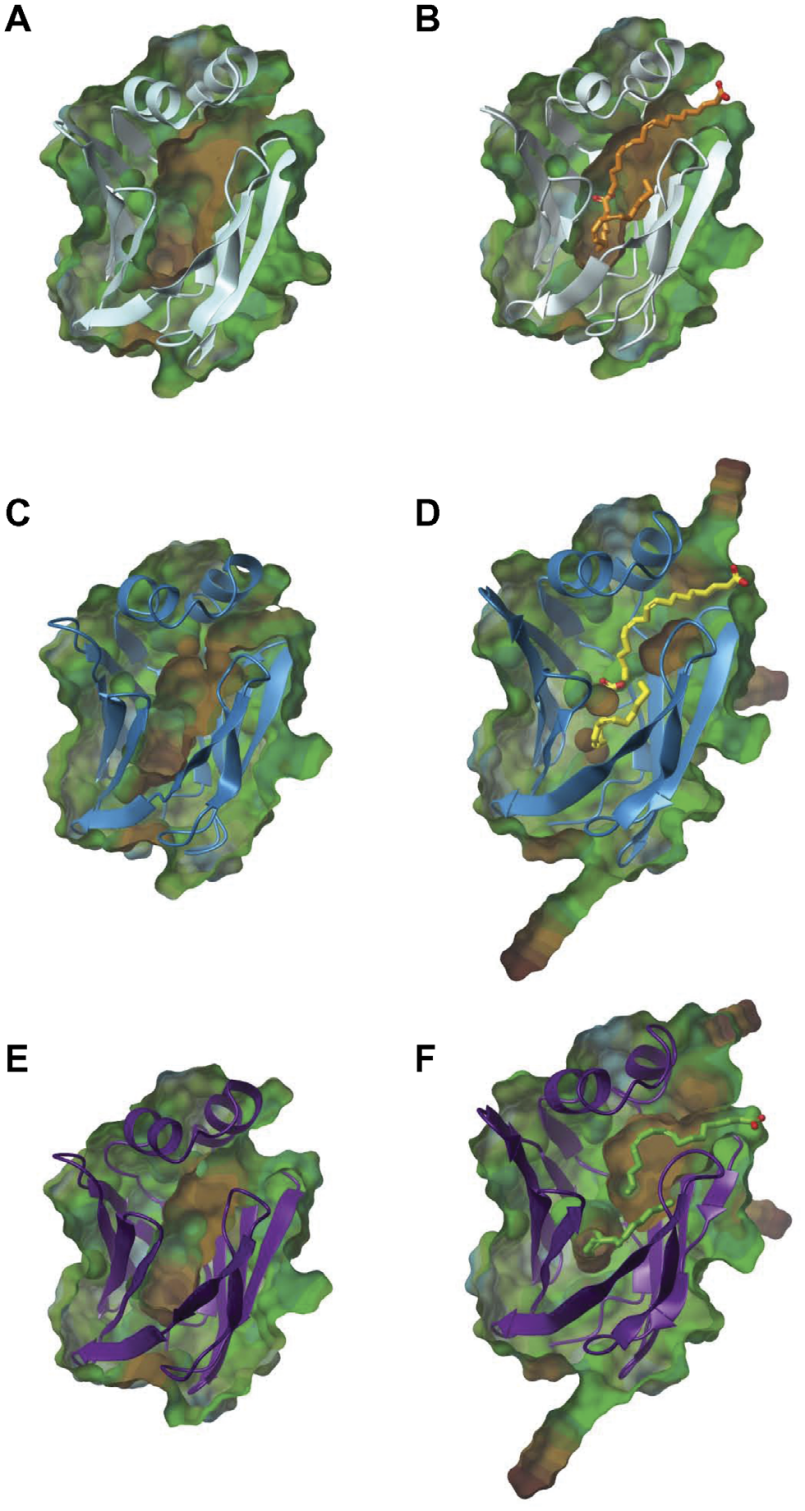
The structural integrity of the binding pocket is significantly affected following 4-HNE adduction. Lipophilic surface maps were utilized to visualize ligand portals and the internal binding cavity of native and 4-HNE adducted L-FABP. Areas colored brown indicate more lipophilic sites of the protein, while the blue indicates more hydrophilic portions. Both apo (A) and holo (B) L-FABP have a well-defined binding area within the protein cavity. Upon 4-HNE adduction, it is clear that both the integrity and size of the binding pocket diminishes in the “open chain” apo (C), holo (D), and the HA structures (E,F).

To further examine the effects of peripheral 4-HNE adduction, Figure 7 shows the lipophilic surface maps that were applied to visualize ligand portals and the internal binding cavity of native and 4-HNE adducted L-FABP. Figure 7A shows that in the apo state, a well-defined ligand entrance site at portal 1 and interior binding cavity exist throughout the β-barrel with an internal binding area of 1,116.38 Å^3^. Figures 7C and E reveal that upon 4-HNE adduction, the binding pocket exhibits diminished solvent accessibility at portal 1 and reduced areas of 634.125 Å^3^ and 597.125 Å^3^, respectively. In the holo state, more dramatic effects of aldehyde adduction are evident, where the binding pocket loses almost all structural integrity. Native holo L-FABP retains an internal binding area of 705 Å^3^ in the presence of two LA ligands as shown by Figure 7B; Figure 7D and F reveal that the 4-HNE modified L-FABP possess areas of 143.375 Å^3^ and 404 Å^3^ with reduced ligand access at portal 1 compared to native holo L-FABP.

**Figure 8:**
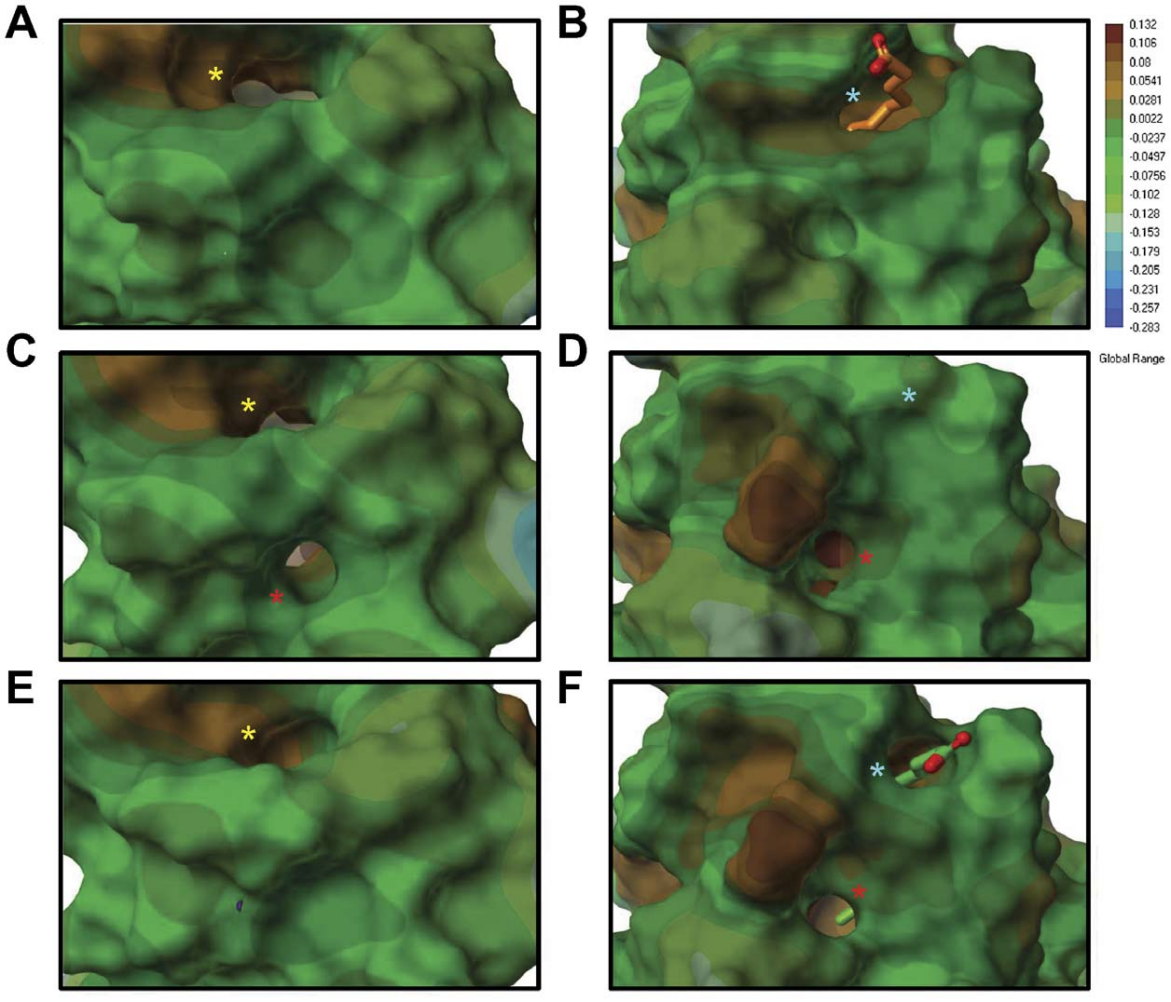
New portals for ligand entry/exit are observed following aldehyde adduction. (B) In the apo state, a new portal appears when 4-HNE is bound below portal 2, P4. In the holo state, a different portal appears slightly south of portal 1, P5, in 4-HNE modified L-FABP (D) and in the 4-HNE hemiacetal (F). Asterisks denote the presence of a portal: Yellow  =  Portal 2; Blue  =  Portal 1; Red  =  P4, P5. Linoleic acid bound in site 2 is also shown in holo-L-FABP: native  =  orange, 4-HNE HA  =  green. The lipophilic map is shown on the right: brown (hydrophobic) to blue scale (hydrophillic) with units given in kcal/mol.

Interestingly, when adducted in the “open chain” form, a new solvent accessible portal (P4, red asterisk) appears below portal 2, between the βG-βH strand interface of apo L-FABP in Figure 8C. In the holo state, an alternative solvent accessible portal was observed at a different position on the protein. When adducted, a new portal also appears below portal 1 between βD-βE as shown in Figure 8D and F (P5, red asterisk). Reference residues around the binding portals were examined at the α carbons to detect changes in the spacial orientation following 4-HNE adduction. These amino acid maps show minimal changes in the 3-dimensional maps surrounding portals 1 and 2 (Figure S3). Therefore, we can attribute these changes in portal solvent accessibility to the changes in the amino acid side chains in the presence of 4-HNE adducts.

## Discussion

Electrophilic products of LPO are reactive molecules that can interact with lipids, proteins and DNA [6]. 4-HNE remains one of the most widely studied aldehydic products of LPO, due to its high prevalence among protein carbonyls and its detection in tissues of patients and animal models of diabetes, cardiovascular disease, chronic obstructive pulmonary disease, nonalcoholic fatty liver disease and ALD [6], [43], [44], [45]. Thus, understanding how protein adduction by reactive aldehydes affects proteins in the cell may aid in elucidating the pathogenesis of a multitude of disease states. The consequences of protein adduction have been reported to affect protein activity, stability and conformation [21], [46], [47], [48], [49], [50]. Currently, protein carbonylation is used as a biomarker for oxidative damage in humans [51], [52]. Therefore, identifying protein targets susceptible to 4-HNE modification *in vivo* may provide mechanistic insight into disease progression. Utilizing proteomics-based approaches, we have begun to identify these proteins following ethanol exposure in mice [19], [20], [21], [53], [54].

Based on previous reports from our laboratory, we have hypothesized that because of its role in in lipid uptake and trafficking, 4-HNE-modified L-FABP may be a contributing factor to the observed accumulation of lipid within hepatocytes in early stages of ALD. This highly expressed protein is severely decreased at the protein level following sustained ethanol consumption and was found through proteomic screens to be immunopositive for 4-HNE [18]. Evidence shows detrimental effects of ethanol consumption on metabolic pathways and 4-HNE adduction of L-FABP may provide a potential mechanism for the observed alterations in hepatic lipid homeostasis [24], [55]. Consistent with previous finding in rats [18], a mouse model of ethanol feeding identified L-FABP as 4-HNE immunopositive, and was associated with a decrease in the FABP1 mRNA and total L-FABP protein pool following ethanol exposure. Other reports have agreed with our results, indicating >50% decrease in the mRNA of FABP1 following a 4 week “ramped” ethanol feeding regimen [56]. However, an earlier publication stating that L-FABP contributes to the increased hepatic cytosolic protein pool in rats following chronic alcohol ingestion contradicts our findings [57]. The interpretation of these data result from less direct measurements of hepatic protein content utilizing gel filtration chromatography and radial immunodiffusion techniques, and may explain the discrepancies between our findings. Lu et al. have also reported that ethanol feeding for 4 weeks in female SV/129 mice had no effect on the levels of L-FABP [58]. It should be noted that this model utilized female mice on a SV/129 background and our studies are conducted on male mice on the C57/Bl6 background – which may explain differences between studies.

Two other members of the lipid binding protein family, adipose (A-FABP) and epithelial (E-FABP) isoforms, are known targets of 4-HNE modification [59], [60]. 4-HNE modified A-FABP has been identified from the adipose tissue of obese mice, and is hypothesized to contribute to insulin resistance [60]. 4-HNE modified E-FABP has also been identified in retinal homogenates of rats and this lipid binding protein is proposed to function as an antioxidant protein by scavenging lipoxidation end-products from the cellular environment [59]. Sequence homologies of A-FABP and E-FABP (∼60%) link the cysteine adducts to be at similar positions within the binding pocket (Cys120 and Cys117); however, L-FABP does not contain this cysteine residue. Identified adducts on L-FABP are prevalent along the periphery of the protein: Cys69, His43 and Lys46 are located at the bottom of the β-barrel near portal 3 at βE, βB and βC respectively; Lys31 and Lys57 surround portal 1 at βD and αII; Lys6 is located on βA of the β-barrel further away from designated portals. Many of the adducted amino acids identified in this study play important roles as constituents of ligand accessible portals **–** indicating that even steric hindrance of these portals by 4-HNE may explain the reduced binding capacity and affinity for ligands. As previously mentioned, L-FABP binds two FA, with the first ligand interacting with residues on the interior of the binding pocket and the carboxyl group of the second ligand interacting directly with Lys31 and Ser56 at the surface of portal 1 [39], [61]. Lys31 and Lys57, amongst other amino acids, constitute Portal 1 and are involved in electrostatic interactions [15], [33], [62], further supporting the potential implications of 4-HNE adduction on these peripheral lysine residues. Due to the nucleophillic nature of some of these targets, it is highly possible that they may have absolutely no contributions to the functionality of the protein. For instance, Lys6 is a peripheral amino acid that is not near the established ligand portals. 4-HNE reacts with the side chains of Cys >> His > Lys residues via MA at C3 or SB reactions at C1. Both of these reactions with 4-HNE were observed on multiple residues of rL-FABP when chemically stabilized. Further analysis of the functional effects of 4-HNE adducts on L-FABP revealed decreased binding, alterations in protein stability, changes in the internal binding pocket and solvent accessible portals. Collectively, these data present evidence for the detrimental effects of 4-HNE modification on L-FABP *in vitro*.

It is difficult to predict the physiological and/or pathological relevance of transient-type lysine-4-HNE adducts *in vivo.* The consequences of reversible modifications are unknown, but may act in a protective manner by reducing irreversible oxidation and modulating protein function [52]. Alternatively, transient 4-HNE adducts on L-FABP could (1) mediate the specificity to bind certain hydrophobic ligands and/or (2) potentially cross-links to other hepatic proteins to regulate their stability/activity. A total of six amino acids were identified as targets of 4-HNE adduction in an *in vitro*-based system. Efforts to identify sites of adduction *in vivo* were conducted using highly sensitive MS instrumentation coupled with L-FABP enrichment strategies, yet were unsuccessful. The techniques employed herein identified L-FABP as a target of 4-HNE adduction; however, sensitivity and specificity may be rate-limiting, further complicating identification of low-abundant *in vivo* protein carbonyls [63]. Additionally, sequence coverage of L-FABP *in vivo* analyses were much less compared to those of the in vitro experiments (34% versus 90%, respectively) and may contribute to the difficulty of identifying specific sites of adduction. Due to limitations in enrichment strategies, it is difficult to speculate on the percentage of adducted L-FABP found in the livers of ethanol-fed mice. Recent findings suggest that highly expressed proteins mask the detection of low-level aldehyde targets [64], [65].

Impacting hepatic uptake and trafficking of lipids through loss of L-FABP protein/function has been demonstrated through the use of L-FABP^−/−^ models [66], [67], [68]. Knock-out mice exhibit decreased FA binding capacity and altered distribution of lipids – with 2–3 fold higher molar ratios of cholesterol/cholesterol ester, cholesteryl ester/triglyceride, and cholesterol/phospholipid [67]. L-FABP deletion inhibits LCFA uptake, reduces LCFA intracellular transport/diffusion and inhibits LCFA esterification and oxidation [17]. In addition, L-FABP gene ablation reduces nuclear distribution of LCFA and peroxisome proliferator-activated receptor-α activity [69], [70]. Capacity to regulate these processes are clearly altered in early ethanol-induced liver injury, and loss of function/translation of L-FABP may explain some of the hepatic alterations in lipid homeostasis.

Equilibrium binding assays revealed changes in affinity and capacity to bind in both sites in a concentration dependent manner, but the effect was not completely inhibitory. When A-FABP was modified with 500 µM 4-HNE a less dramatic effect was observed, exhibiting a K_d_ of 23.20 µM when modified compared to a Kd of 2.05 µM of the native protein [60]. It should be noted that these concentrations of 4-HNE are above what are thought to be involved in pathological disease states. Basal concentrations of 4-HNE in many tissues and serum are predicted to be below 0.1 µM and can range from 3.8 µM up to 100 µM in a stressed liver [6], [71], [72]. 4-HNE treatments in our studies were maintained within physiological and pathological concentrations of 4-HNE to retain relevance to disease pathogenesis. In the present study, non-linear regression analysis showed that very low concentrations of 4-HNE actually increased affinity toward ANS in the primary binding site. These data suggest that lower concentrations of 4-HNE may provoke a regulatory effect on L-FABP ligand binding and mediate the specificity of ligands and their respective destinations to various cellular compartments. It is established that two factors confer selectivity towards ligands (1) increased hydrophobicity of the ligand (2) and the electrostatic interactions of the surface residues around portals [73], [74]. In addition to recognizing that steric hindrance around the portals may be contributing to decreased binding ability, changes in the electrostatic interactions of proximal residues (i.e. lysine residues) due to 4-HNE adduction (similar to previously published reports [75], [76]) may contribute the alterations in ligand specificity that we observe in our *in vitro* experiments. 4-HNE adduction also lowers affinity for SA and LA, but increases affinity for OA. It was observed with all FA displacement curves that the total capacity to bind was severely diminished following 4-HNE adduction. The results from these displacement assays provides further evidence that these pathological concentrations of 4-HNE have the potential to mediate ligand specificity within the cell.

It has been well documented that post-translational modification of proteins, including 4-HNE adduction, affects conformational stability and function [11], [18]. We have indirectly assessed the stability of L-FABP *in vivo* by quantifying the amount of ubiquitinated protein in response to the ethanol diet. The ubiquitin system targets hydrophobic lysine residues exposed on denatured protein, providing mechanistic insight to global protein stability [77]. Ethanol increases the amount of poly-ubiquitinated L-FABP, further supporting our *in vitro* denaturation studies *in vivo*. The stability of rL-FABP following 4-HNE adduction is highly regulated by the binding state of the protein. In the apo form, 4-HNE adduction was detrimental to protein stability in a concentration dependent manner; however, holo rL-FABP adducted with 4-HNE revealed the aldehyde had modest effects on global protein stability. Modification of E-FABP with 4-HNE showed similar effects on protein stability, assessed via chemical denaturation with guanidine hydrochloride in the apo state [59]. Binding of substrates and/or cofactors have been shown to increase the thermostability of proteins, demonstrated with heat shock protein 70 [78]. It is unknown whether 4-HNE modified L-FABP in the holo state is a highly prevalent species and future studies are in order to fully understand the altered stability and these implications *in vivo*.

Changes in global L-FABP structure resulting from 4-HNE modification were assessed *in silico*. Protein conformational shifts can be attributed to several important physiological processes, including (but not limited to) cofactor/substrate docking, solvent pH, and post-translational modifications like 4-HNE adduction. 4-HNE adducted apo and holo L-FABP minimized structures revealed slight but minimal differences in global conformation compared to the native protein, as shown by the RMSD values. This was also demonstrated by modest changes in the difference in distance of the alpha carbons of reference residues between native and 4-HNE modified L-FABP. These homology models show variations in the orientations of the bound LA in each binding site. We also observed reductions in the internal binding pocket of both apo and holo L-FABP following 4-HNE adduction. These reduced areas may also confer selectivity for less bulky ligands (instead of heme, bile salts, cholesterol, etc.) and favor smaller, more flexible compounds. Considering this possibility, L-FABP may function more like intestinal FABP (I-FABP). It has an internal binding pocket approximately half that of L-FABP (234 Å^3^ versus 440 Å^3^) and specifically binds LCFA at a 1∶1 ratio, with lower affinities for unsaturated FA [15], [33].

Solvent mapping around the binding portals revealed substantial changes in established portal accessibility and formation of new solvent accessible portals throughout L-FABP structure, regardless of binding state. These results indicate that the functional dynamics that regulate L-FABP may be attributed to the side-chains of a few peripheral amino acids surrounding the binding portals. L-FABP may also be adapting to these modifications and still functioning to bind lipids, just at a reduced capacity. It is unknown whether other post-translational modifications (PTM) exhibit similar effects on predicted ligand portals. When Cys69 is glutathionylated, the binding profile is not altered; but the modified species is more susceptible to proteolysis [79], [80]. It has been reported that acetylation of surface lysine residues does not change tertiary structure nor contribute to altered binding properties [81], [82]. This PTM, however, cannot be compared to 4-HNE; there is a greater effect of steric hindrance from the longer chain length of the aldehyde. In summary, this report has demonstrated the dynamic processes involved with 4-HNE adduction of L-FABP, providing new evidence for cellular perturbations in lipid trafficking and metabolism observed with early stages of ALD.

## Supporting Information

Figure S1 **4-HNE adducts are identified on peptides AIGLPEDLQK_31HNE_GK, GVSEIVH_43HNE_EGK, and LTITYGPK_57HNE_VVR.** All of these protein adducts were identified to react with 4-HNE through a MA-type reaction. Identified b/y ions are labeled within the spectrum and shown along the peptide backbone above the spectrum. The parent ion of the MS/MS fragment is shown as an inset in the upper right corner of the spectrum.
(TIF)

Figure S2 **Schiff base adducts were identified on peptides LTITYGPK_57HNE_VVR (A and B) and MNFSGK_6HNE_YQLQSQENFEPFMK (C and D) of L-FABP.** Spectra are shown for the same peptide, with varying degrees of methionine oxidation. Identified b/y ions are labeled within the spectrum and shown along the peptide backbone above the spectrum. The parent ion of the MS/MS fragment is shown as an inset in the upper right corner of the spectrum.
(TIF)

Figure S3 **Alpha carbons of reference residues that surround binding portals 1 and 2 were mapped to visualize changes in ligand accessibility following 4-HNE adduction.** Native apo (A and D) and holo (G and J); 4-HNE adducted apo (B and E) and holo (H and K), and 4-HNE HA apo (C and F) and holo (I and L).
(TIF)

Table S1 **4-HNE immunopositive proteins picked and identified in cytosolic fractions of mice chronically fed with ethanol.**
(DOC)
