# Supplementary figures and images for: Characterization of 4-HNE Modified L-FABP Reveals Alterations in Structural and Functional Dynamics
Source: PLoS One. 2012 Jun 6;7(6):e38459. doi: 10.1371/journal.pone.0038459 (PMC3368874; doi:10.1371/journal.pone.0038459)

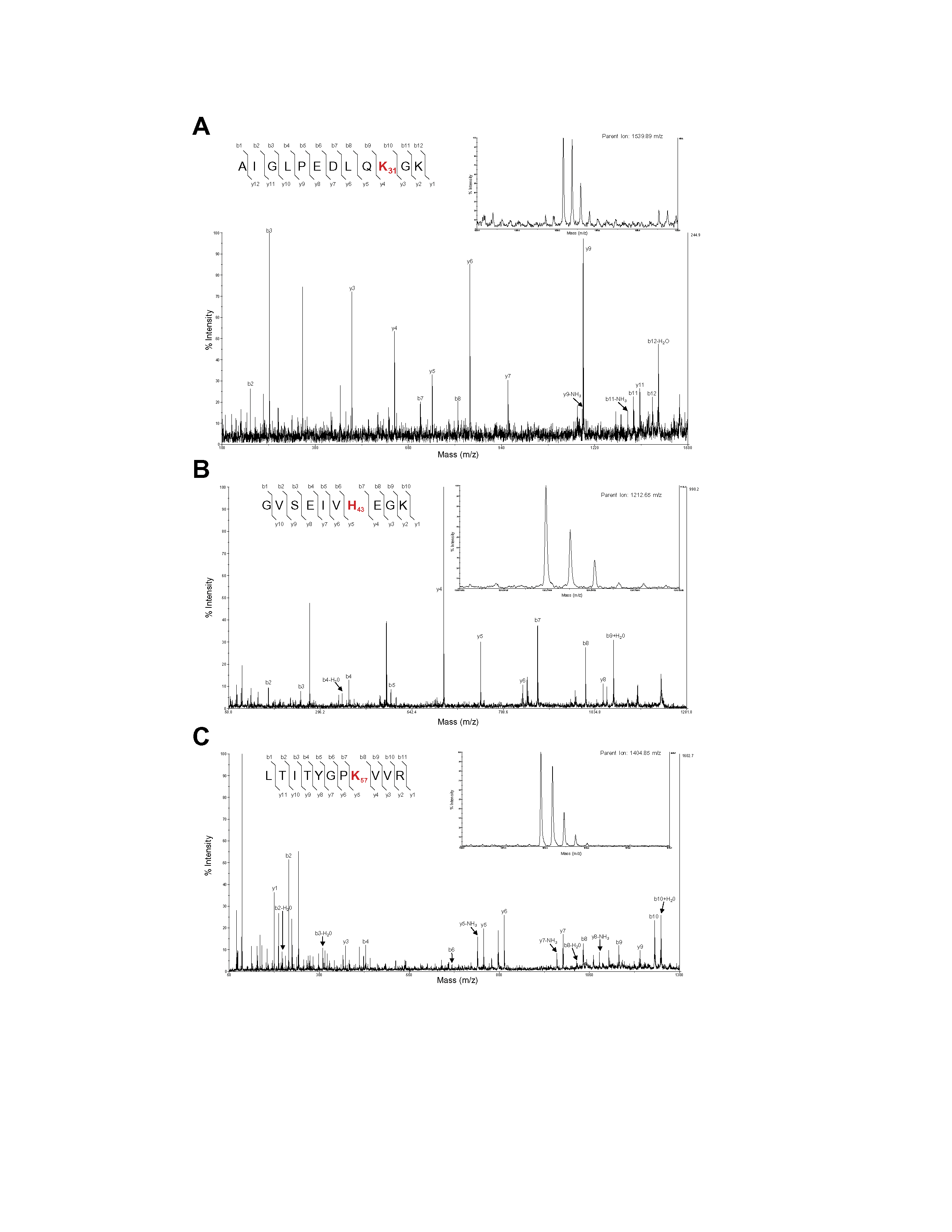

Supplement: Figure S1 — 4-HNE adducts are identified on peptides AIGLPEDLQK31HNEGK, GVSEIVH43HNEEGK, and LTITYGPK57HNEVVR. All of these protein adducts were identified to react with 4-HNE through a MA-type reaction. Identified b/y ions are labeled within the spectrum and shown along the peptide backbone above the spectrum. The parent ion of the MS/MS fragment is shown as an inset in the upper right corner of the spectrum. (TIF) [file pone.0038459.s001.tif]

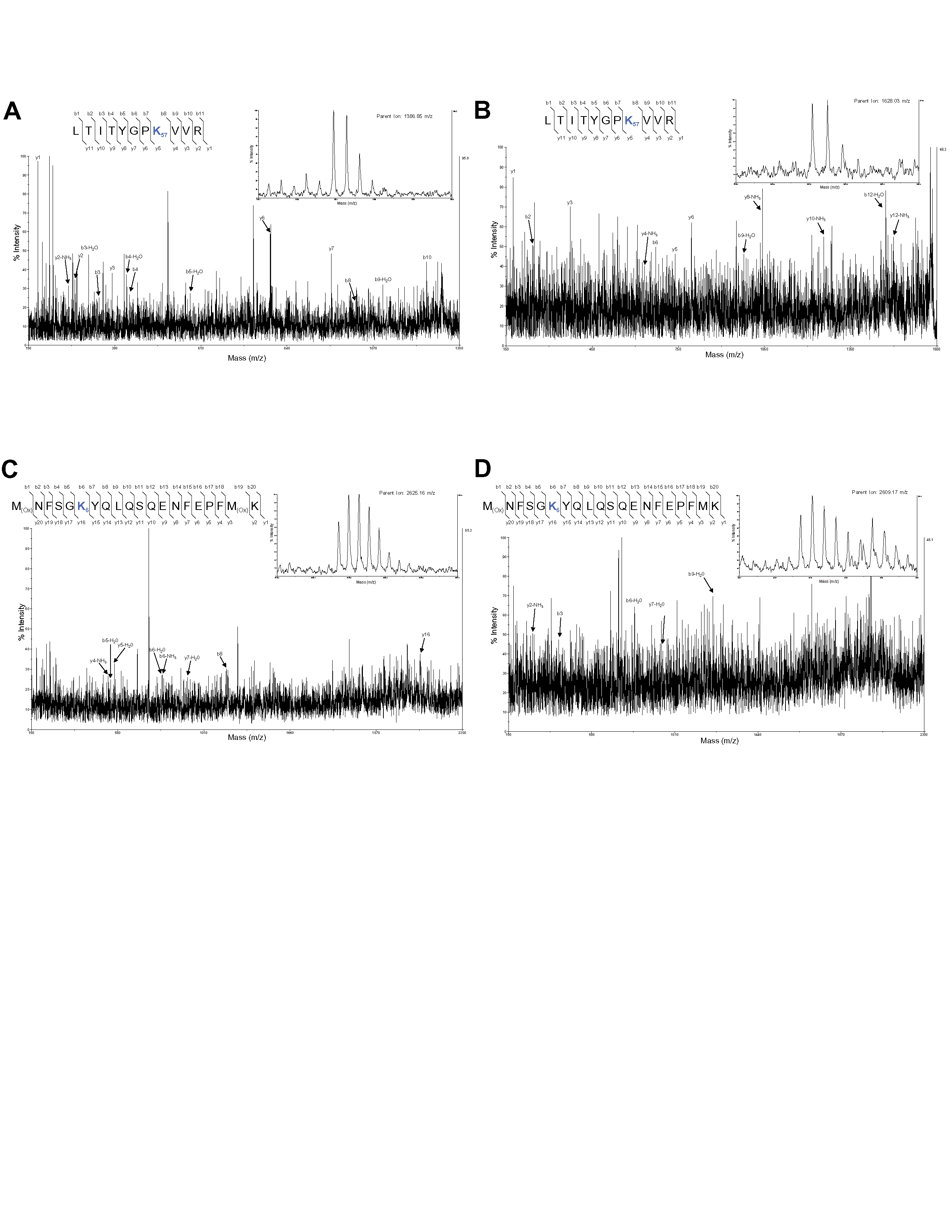

Supplement: Figure S2 — Schiff base adducts were identified on peptides LTITYGPK57HNEVVR (A and B) and MNFSGK6HNEYQLQSQENFEPFMK (C and D) of L-FABP. Spectra are shown for the same peptide, with varying degrees of methionine oxidation. Identified b/y ions are labeled within the spectrum and shown along the peptide backbone above the spectrum. The parent ion of the MS/MS fragment is shown as an inset in the upper right corner of the spectrum. (TIF) [file pone.0038459.s002.tif]

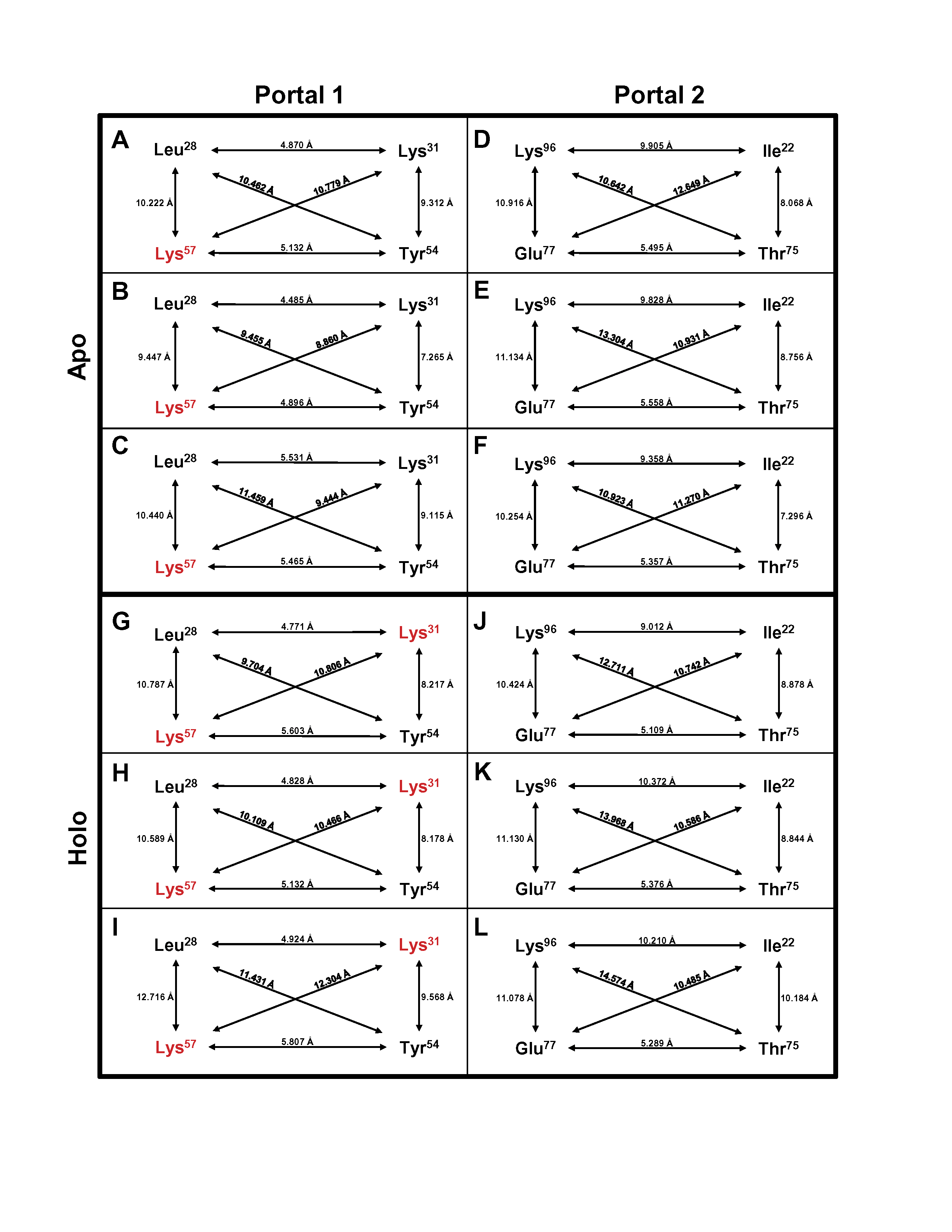

Supplement: Figure S3 — Alpha carbons of reference residues that surround binding portals 1 and 2 were mapped to visualize changes in ligand accessibility following 4-HNE adduction. Native apo (A and D) and holo (G and J); 4-HNE adducted apo (B and E) and holo (H and K), and 4-HNE HA apo (C and F) and holo (I and L). (TIF) [file pone.0038459.s003.tif]
