# Supplementary material for: Characterization of 4-HNE Modified L-FABP Reveals Alterations in Structural and Functional Dynamics
Source: PLoS One. 2012 Jun 6;7(6):e38459. doi: 10.1371/journal.pone.0038459 (PMC3368874; doi:10.1371/journal.pone.0038459)
Supplement: Table S1 — 4-HNE immunopositive proteins picked and identified in cytosolic fractions of mice chronically fed with ethanol. (DOC) [file pone.0038459.s004.doc]

Table S1. 4-HNE immunopositive proteins picked and identified in cytosolic fractions of mice chronically fed with ethanol.

| Spot | Accession ID* | Common Name | % Coverage | MASCOT Score | Peptides |
| --- | --- | --- | --- | --- | --- |
| 1 | SODC_MOUSE | SOD1 | 32 | 199 | 4 |
| 2 | DOPD_MOUSE | D-dopachrome decarboxylase | 95 | 315 | 9 |
| 3 | HBB1_MOUSE | Hemoglobin subunit beta-1 | 70 | 311 | 9 |
| 4 | IPYR_MOUSE | Inorganic Phosphatase | 52 | 432 | 13 |
|  | ANXA4_MOUSE | Annexin A4 | 28 | 245 | 8 |
| 5 | KHK_MOUSE | Ketohexokinase | 8 | 60 | 2 |
| 6 | NO ID |  |  |  |  |
| 7 | ANXA5_MOUSE | Annexin A5 | 51 | 545 | 16 |
| 8 | FABPL_MOUSE | Fatty acid-binding protein, liver | 34 | 303 | 4 |
|  | ACTBL_MOUSE | Actin (Beta-Actin-like protein 2) | 14 | 148 | 4 |
| 9 | A1AT1_MOUSE | Alpha-1-antitrypsin 1-1 | 21 | 219 | 7 |
| 10 | A1AT4_MOUSE | Alpha-1-antitrypsin 1-4 | 31 | 319 | 11 |
| 11 | A1AT1_MOUSE | Alpha-1-antitrypsin 1-1 | 18 | 277 | 8 |
| 12 | NO ID |  |  |  |  |
| 13 | A1AT1_MOUSE | Alpha-1-antitrypsin 1-1 | 21 | 240 |  |
|  | A1AT4_MOUSE | Alpha-1-antitrypsin 1-4 | 16 | 198 | 6 |
|  | A1AT5_MOUSE | Alpha-1-antitrypsin 1-5 | 12 | 136 | 4 |
| 14 | HSP7C_MOUSE | HSP 70 (Heat Shock Cognate 71 kDa) | 44 | 995 | 26 |
|  | ANXA6_MOUSE | Annexin A6 | 21 | 356 | 14 |
|  | ALBU_MOUSE | Serum Albumin | 5 | 64 | 3 |
| 15 | ALBU_MOUSE | Serum Albumin | 54 | 1007 | 28 |
| 16 | ADK_MOUSE | Adenosine Kinase | 47 | 450 | 15 |
|  | ANXA7_MOUSE | Annexin A7 | 22 | 274 | 9 |
|  | METK1_MOUSE | S-adenosylmethionine synthase isoform type 1 | 16 | 212 | 6 |
| 17 | ADK_MOUSE | Adenosine Kinase | 26 | 342 | 10 |
| 18 | NO ID |  |  |  |  |
| 19 | F16P1_MOUSE | Fructose-1,6-bisphosphatase 1 | 53 | 575 | 14 |
|  | MDHC_MOUSE | Malate dehydrogenase, cytoplasmic | 26 | 242 | 9 |
|  | DHDH_MOUSE | Trans-1,2-dihydrobenzyne-1,2-dioldehydrogenase | 28 | 233 | 9 |
| 20 | PSA1_MOUSE | Protease subunit alpha type-1 | 35 | 218 | 9 |
| 21 | NIT2_MOUSE | Omega-amidase NIT2 | 42 | 273 | 9 |
|  | PNPO_MOUSE | Pyridoxine-5'-phophate oxidase | 12 | 101 | 3 |
|  | MAAI_MOUSE | Maleylacetoacetate isomerase | 17 | 72 | 3 |
| 22 | PGAM1_MOUSE | Phosphoglycerate mutase 1 | 42 | 275 | 8 |
|  | CAH3_MOUSE | Carbonic Anhydrase 3 | 33 | 248 | 8 |
|  | ESTD_MOUSE | S-formylglutathione hydrolase | 36 | 150 | 7 |
| 23 | CAH3_MOUSE | Carbonic Anhydrase 3 | 45 | 284 | 9 |
| 24 | NO ID |  |  |  |  |
| 25 | CAH3_MOUSE | Carbonic Anhydrase 3 | 37 | 354 | 8 |
| 26 | CAH3_MOUSE | Carbonic Anhydrase 3 | 58 | 384 | 12 |
|  | DHPR_MOUSE | Dihydropteridine reductase | 44 | 197 | 7 |
| 27 | IDHC_MOUSE | Isocitrate Dehydrogenase [NADP] cytoplasmic | 57 | 772 | 23 |
|  | HPPD_MOUSE | 4-Hydroxyphenylpyruvate dioxygenase | 19 | 296 | 7 |
|  | PGK1_MOUSE | Phosphoglycerate Kinase 1 | 17 | 175 | 6 |
|  | FAAA_MOUSE | Fumarylacetoacetase | 10 | 175 | 4 |
| 28 | AATC_MOUSE | Aspartate aminotransferase | 48 | 556 | 18 |
|  | FAAA_MOUSE | Fumarylacetoacetase | 13 | 149 | 5 |
|  | HPPD_MOUSE | 4-Hydroxyphenylpyruvate dioxygenase | 13 | 120 | 4 |
| 29 | AK1A1_MOUSE | Alcohol Dehydrogenase [NADP+] | 33 | 286 | 10 |
|  | ADHX_MOUSE | Alcohol Dehydrogenase (class-3) | 12 | 102 | 5 |
| 30 | NO ID |  |  |  |  |
| 31 | PGK1_MOUSE | Phosphoglycerate Kinase 1 | 46 | 490 | 15 |
|  | AATC_MOUSE | Aspartate aminotransferase, cytoplasmic | 15 | 186 | 6 |
| 32 | BHMT1_MOUSE | Betaine-homocysteine S-methyltransferase 1 | 38 | 405 | 12 |
|  | ASSY_MOUSE | Arginosuccinate Synthase | 7 | 167 | 5 |
|  | PGK1_MOUSE | Phosphoglycerate Kinase 1 | 17 | 142 | 6 |
| 33 | PGK1_MOUSE | Phosphoglycerate Kinase 1 | 56 | 645 | 18 |
|  | ASSY_MOUSE | Arginosuccinate Synthase | 20 | 268 | 9 |
|  | BHMT1_MOUSE | Betaine-homocysteine S-methyltransferase 1 | 16 | 178 | 6 |
| 34 | GRP78_MOUSE | 78 kDa glucose-regulated protein | 26 | 444 | 15 |
|  | RGN_MOUSE | Regucalcin | 28 | 230 | 7 |
|  | TGM2_MOUSE | Protein-glutamine gamma-glutamyltransferase 2 | 3 | 188 | 7 |
| 35 | PCKGC_MOUSE | Phophoenolpyruvate Carboxykinase, cytosolic [GTP] | 11 | 188 | 7 |
| 36 | PCKGC_MOUSE | Phophoenolpyruvate Carboxykinase, cytosolic [GTP] | 34 | 520 | 17 |
| 37 | DHAK_MOUSE | Bifunctional ATP-dependent dihydroxyacetone kinase/FAD-AMP lyase (cyclizing) | 30 | 494 | 14 |
|  | PGM1_MOUSE | Phosphoglucomuctase 1 | 19 | 282 | 11 |
|  | CBS_MOUSE | Cystathionine beta-synthase | 17 | 206 | 9 |
| 38 | DHAK_MOUSE | Bifunctional ATP-dependent dihydroxyacetone kinase/FAD-AMP lyase | 53 | 865 | 20 |
|  | STIP1_MOUSE | Stress-induced phosphoprotein 1 | 24 | 371 | 13 |
|  | PGM1_MOUSE | Phosphoglucomutase-1 | 25 | 338 | 13 |
|  | CBS_MOUSE | Cystathionine beta-synthase | 11 | 138 | 6 |
|  | PUR9_MOUSE | Bifunctional purine biosynthesis protein PURH | 8 | 81 | 4 |
| 39 | DHAK_MOUSE | Bifunctional ATP-dependent dihydroxyacetone kinase/FAD-AMP lyase (cyclizing) | 10 | 166 | 5 |

*Significant protein match based on -10*LogP MOWSE score of 84 (p < 0.05). Minimal score of significance is 54
